# O025. Excitability of the motor cortex in migraine changes with the distance from the last attack

**DOI:** 10.1186/1129-2377-16-S1-A158

**Published:** 2015-09-28

**Authors:** Gianluca Coppola, Francesca Napoli, Davide Di Lenola, Martina Bracaglia, Mariano Serrao, Cherubino Di Lorenzo, Francesco Pierelli

**Affiliations:** 1 Department of Neurophysiology of Vision and Neuro-ophthalmology, G.B. Bietti Foundation IRCCS, Rome, Italy; 2 Department of Medico-Surgical Sciences and Biotechnologies, “Sapienza” University of Rome Polo Pontino, Latina, Italy; 3 Don Carlo Gnocchi Onlus Foundation, Milan, Italy

## Background

Single-pulse transcranial magnetic stimulation studies of motor cortex have the advantage of relying on an objective measure, the motor evoked potential (MEP) recorded in peripheral muscles, to non-invasively explore the cortical excitability. Previously, thresholds for MEP were found to be normal, increased or even reduced in migraine. In the present study, we investigated whether the level of cortical excitability changes with the distance from the last migraine attack could explain these inconsistent results.

## Methods

Twenty-six patients with untreated migraine without aura (MO) underwent MEP study between attacks and were compared to a group of 24 healthy volunteers (HV). The TMS figure-of-eight coil was positioned over the left motor area. We first identified the resting motor threshold (RMT) and then amplitude of MEP was evaluated by delivering and averaging 10 single pulses of TMS using a stimulus intensity of 120% RMT at a rate of 0.1 Hz.

## Results

Mean RMTs (54.2 in MO vs. 55.8 in HV) and MEP amplitudes (3057 V in MO vs. 3675 V in HV) were not significantly different between MO and HV. In MO, the RMT negatively correlated with days elapsed since the last migraine attack (r = -0.426, p = 0.03).

## Conclusions

From the present data emerges that the threshold for evoking MEP is influenced by the proximity of an attack since it is minimal at a long time interval after an attack, while it is greater and within the range of normative values approaching an attack. The dynamic RMT variations found here resemble those we had previously reported for visual and somatosensory evoked potentials, and may represent time-dependent plastic changes in brain excitability in relation with the migraine cycle.

Written informed consent to publish was obtained from the patient(s).

